# Supplementary material for: Unbiased choice of global clustering parameters for single-molecule localization microscopy
Source: Sci Rep. 2022 Dec 29;12:22561. doi: 10.1038/s41598-022-27074-1 (PMC9800574; doi:10.1038/s41598-022-27074-1)
Supplement: Supplementary file 1 — Supplementary Information. [file 41598_2022_27074_MOESM1_ESM.pdf]

# Supplemental Information

| Publication | SMLM method | Target molecule | minPts | r | Rationale |
|---|---|---|---|---|---|
| Endesfelder et al. [13] (2013) | PALM | RNA Polymerase | 4 | 30nm | *minPts* as recommended in [19] |
| Virant et al. [37] (2018) | dSTORM | various targets | 6 | 40nm | refer to Ref. [13] |
| Sanchez et al. [38] (2019) | PALM | VAR2CSA protein | 5 | 30nm | - |
| Shrivastava et al. (2019) [39] | STORM | Exogenous fibrillar Tau | 20 | 20nm | - |
| Shrivastava et al. (2020) [40] | STORM | fibrillar a-Syn polymorphs | 20 | 20nm | - |
| Mayr et al. (2020) [50] | 3D dSTORM | CD41&CD62p proteins | 3 | 2×davg | Tuned to filter noise |
| Mayr et al. (2020) [50] | 3D dSTORM | CD41&CD62p proteins | 5 | 1.5×davg | Tuned for cluster assignment |
| Harwardt et al. (2020) [16] | DNA-PAINT | MET and epidermal growth factor receptor (EGFR) | 10,15 | 10-15nm | nearest neighbor-based analysis (NeNA) |

Table S1: Overview of hyperparameters used for DBSCAN for the analysis of SMLM datasets. An overview of all clustering methods can be found in Ref. [24]. davg is defined as the average of the minimum distance between two points.

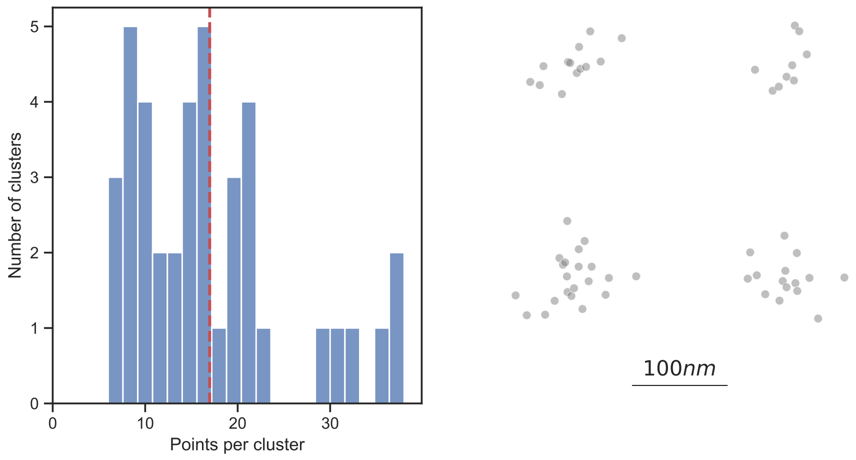


(a) Statistics and samples of 40 clusters extracted from SRM of a neuronal dendrite [9]. Largest cluster diameter: 150$nm$. Mean number of localizations per cluster: 17 (red dashed line).

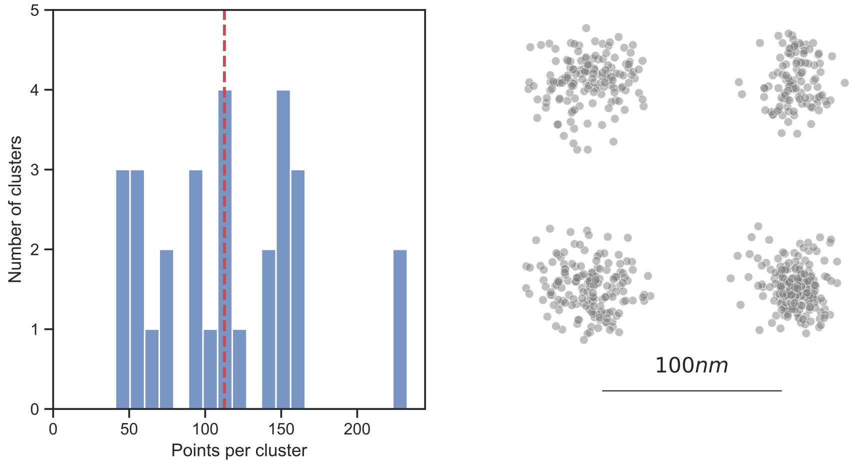


(b) Statistics and samples of 29 Clusters extracted from a DNA Origami trimer dataset. Largest cluster diameter: 77$nm$. Mean number of localizations per cluster: 113 (red dashed line).

Figure S1: **Cluster libraries:** Size distribution (left) and four samples (right) of two libraries of clusters used in this work.

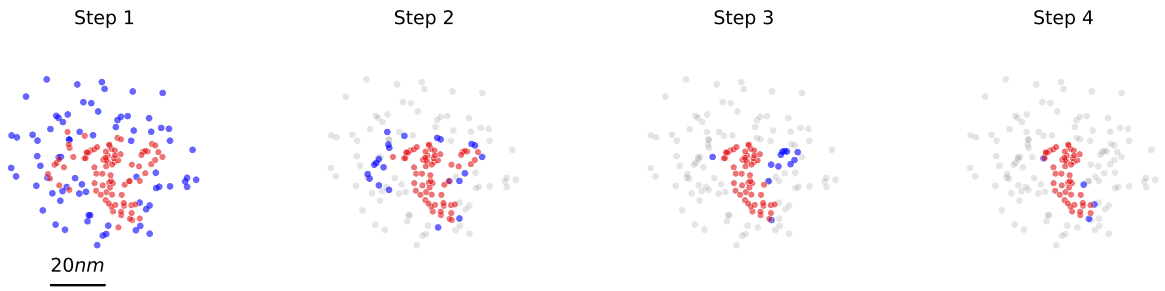


Figure S2: Example of the first four iterative steps of noise removal in DBSCAN (noise free). Points that have the minimum number of neighboring points $minPts = 10$ within a given distance $\varepsilon = 7nm$ are considered as core points (shown in red). Iteratively, all points which do not satisfy this condition are removed (shown in blue), therefore restricting the number of spurious inclusions. Points that have been excluded in previous iterations are shown in grey.

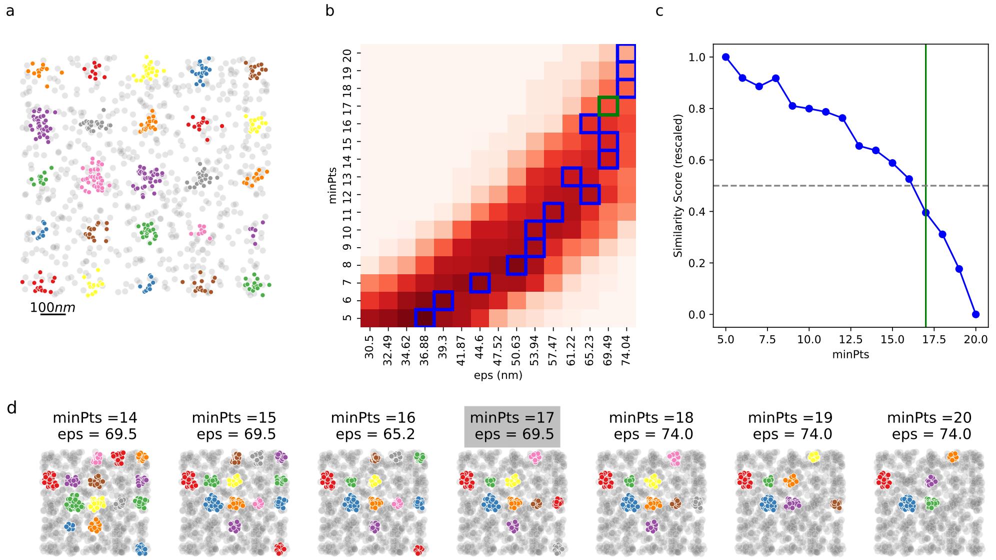


(a) Similarity-based hyperparameter finding with DBSCAN (noisefree)

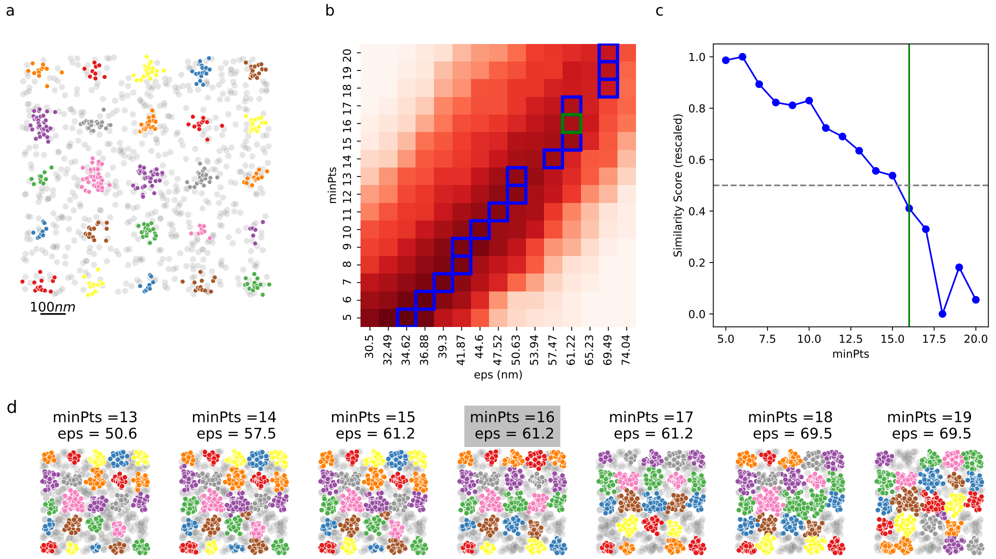


(b) Similarity-based hyperparameter finding with DBSCAN

Figure S3: FINDER applied to configuration shown in Fig. 4(a) of the main manuscript. **Top**: DBSCAN (noisefree). **Bottom**: DBSCAN. **a**:Ground-truth clusters are shown in color, and random noise localizations in grey. **b**:The similarity score as a function of the parameters. See "FINDER algorithm" in "Methods" for a description. A darker red indicates an higher value of similarity. The blue squares highlights the points selected as the *line of optima* (step 6 of the "FINDER algorithm"). The green squares indicates parameters selected by FINDER. **c**:The parameters selection. As described in step 7 of "FINDER algorithm", the final parameter configuration (green vertical line) on the *line of optima* is selected as the first one to fall under the threshold $\alpha$ (dashed horizontal line). Here $\alpha = 0.5$. **d**:Clustering results for the optimal value of epsilon (grey background), and the three lower and higher values within the *line of optima*. See Fig. S19 for an analysis of the variation of the number of $\varepsilon$-values within the interval of interest.

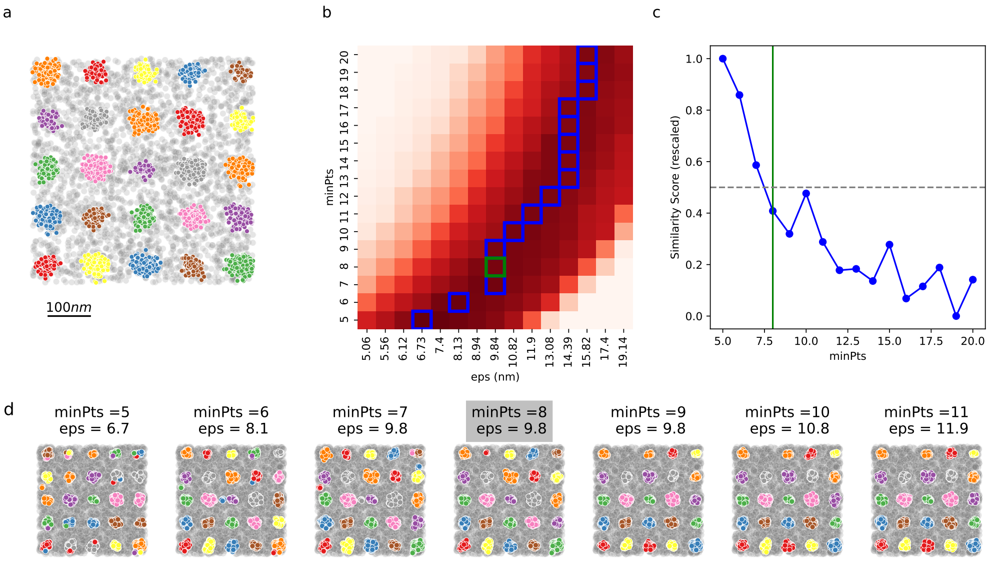


(a) Similarity-based hyperparameter finding with DBSCAN (noisefree)

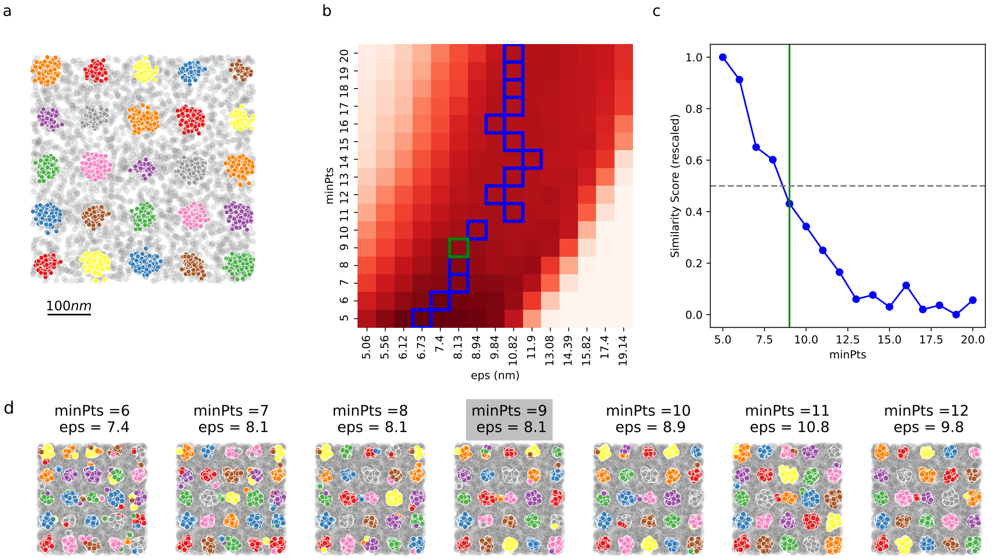


(b) Similarity-based hyperparameter finding with DBSCAN

Figure S4: FINDER applied to configuration shown in Fig. 4(d) of the main manuscript. For details, see caption of Fig. S3. See Fig. S19 for an analysis of the variation of the number of $\varepsilon$-values within the interval of interest.

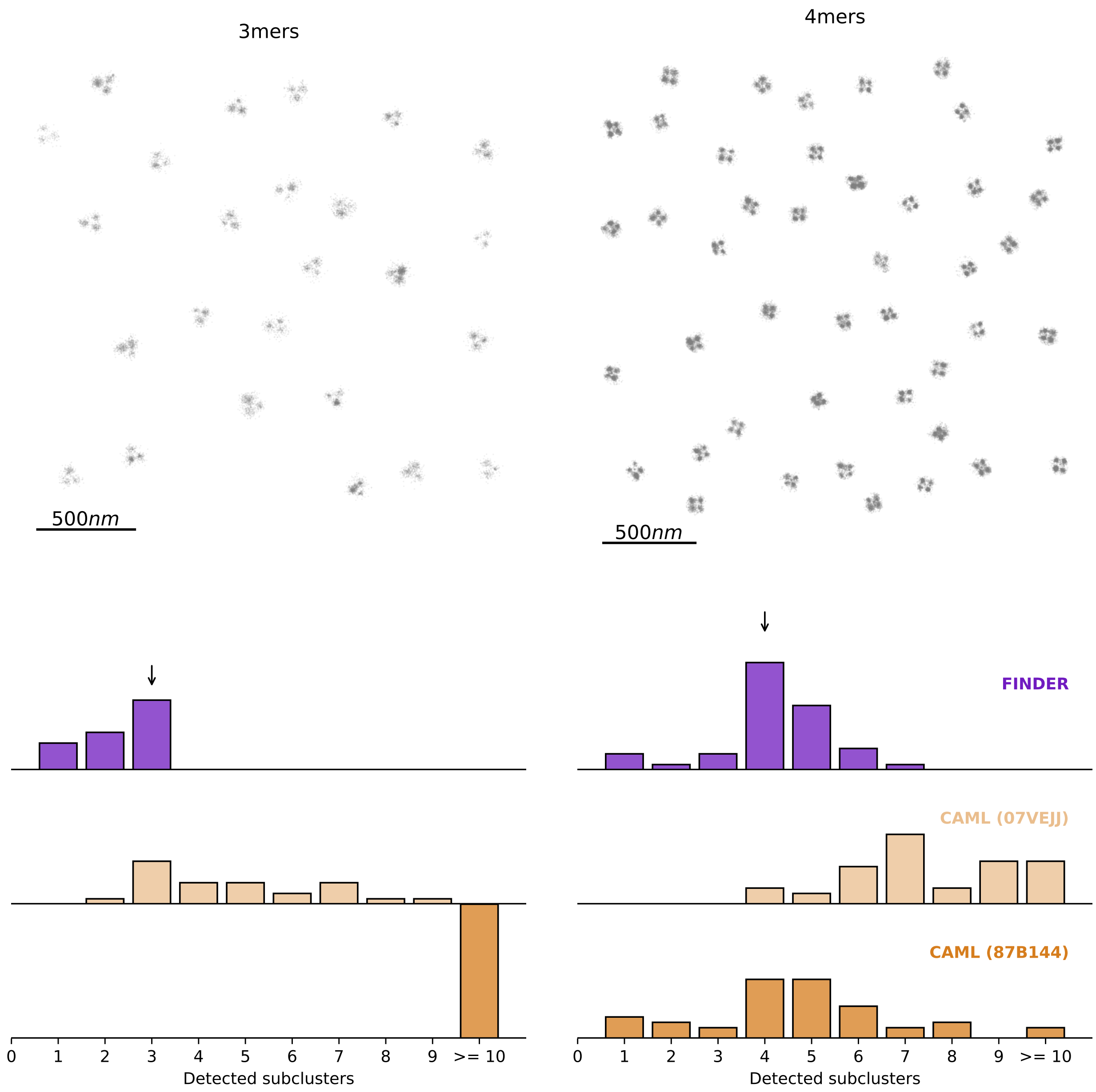


Figure S5: Relates to Fig. 3. Same computation as in Fig. 3, but without added random noise. Here, the optimal radial parameters identified by FINDER for DBSCAN (noisefree) are $\varepsilon = 8.99$ and $minPts = 10$ (trimer), and $\varepsilon = 3.83$ and $minPts = 8$ (tetramer). 3-mers are not recognized by CAML (87B144).

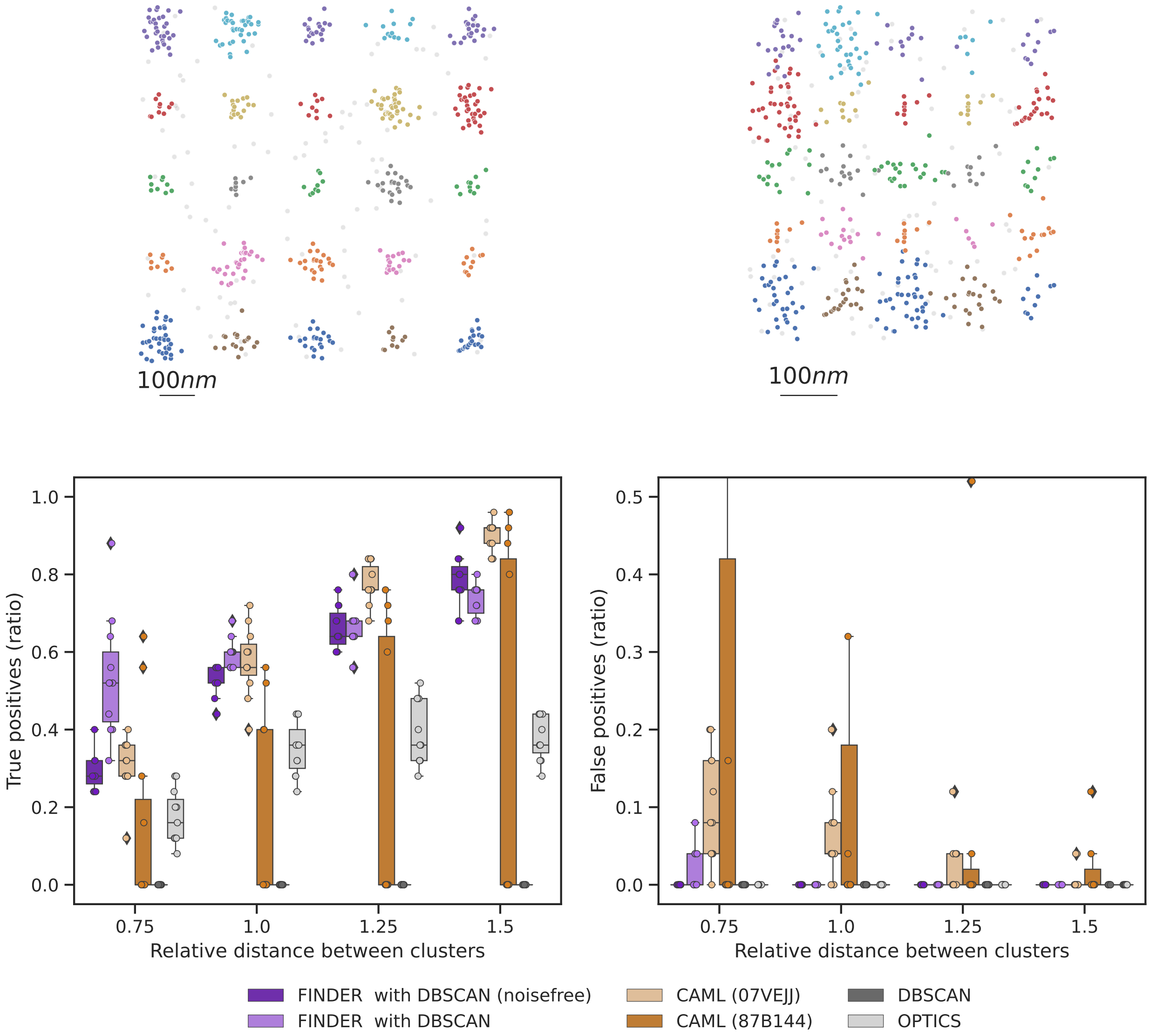


Figure S6: Relates to Fig. 4 a of the main manuscript. Clusters from a SMLM dataset of a synapse [9] (see Fig. 1(a)) are randomly assigned to a $5 \times 5$ grid. Based on the number of localizations, a ratio of 0.2 of random noise localizations was then added to the domain. The spacing in between clusters was decreased, here shown as multiples of the maximal cluster diameter of the cluster library, here $150nm$. Top row: Two samples with highest and lowest spacing. Boxplots show the number of true and false positive cluster detections. Parameters used for DBSCAN [19] were $\varepsilon = 10$nm, minPts $= 10$ and for OPTICS[22]: minPts $= 20$ , xi $= 0.05$, max epsilon $= 100$ nm.

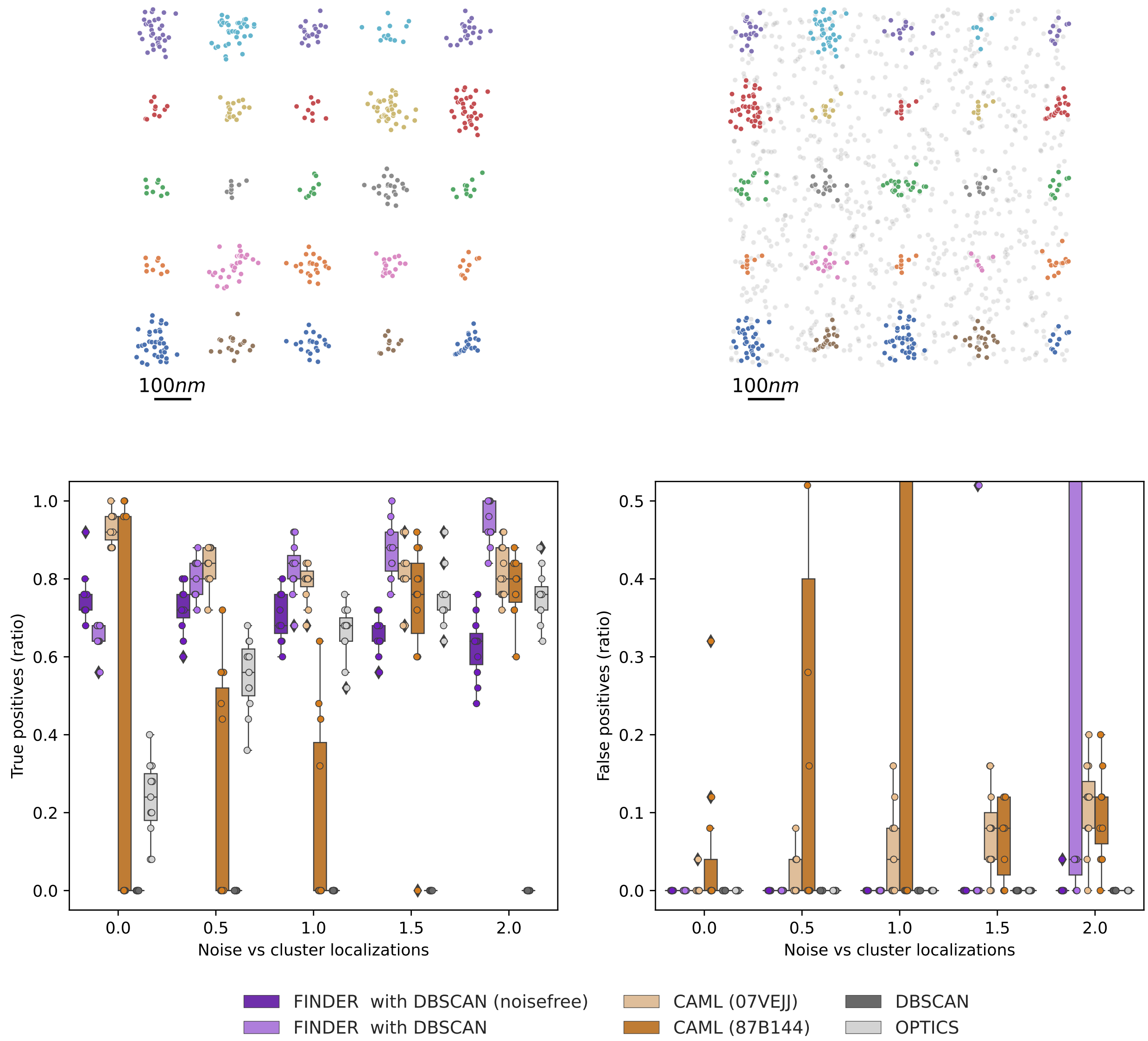


Figure S7: Relates to Fig. 4 b of the main manuscript. Clusters from a SMLM dataset of a synapse [9] (see Fig. 1(a)) are randomly assigned to a $5 \times 5$ grid, with spacing $225\mu$m. Based on the number of localizations, an increasing ratio of random noise localizations was then added to the domain. See caption of Fig. S6 for details.

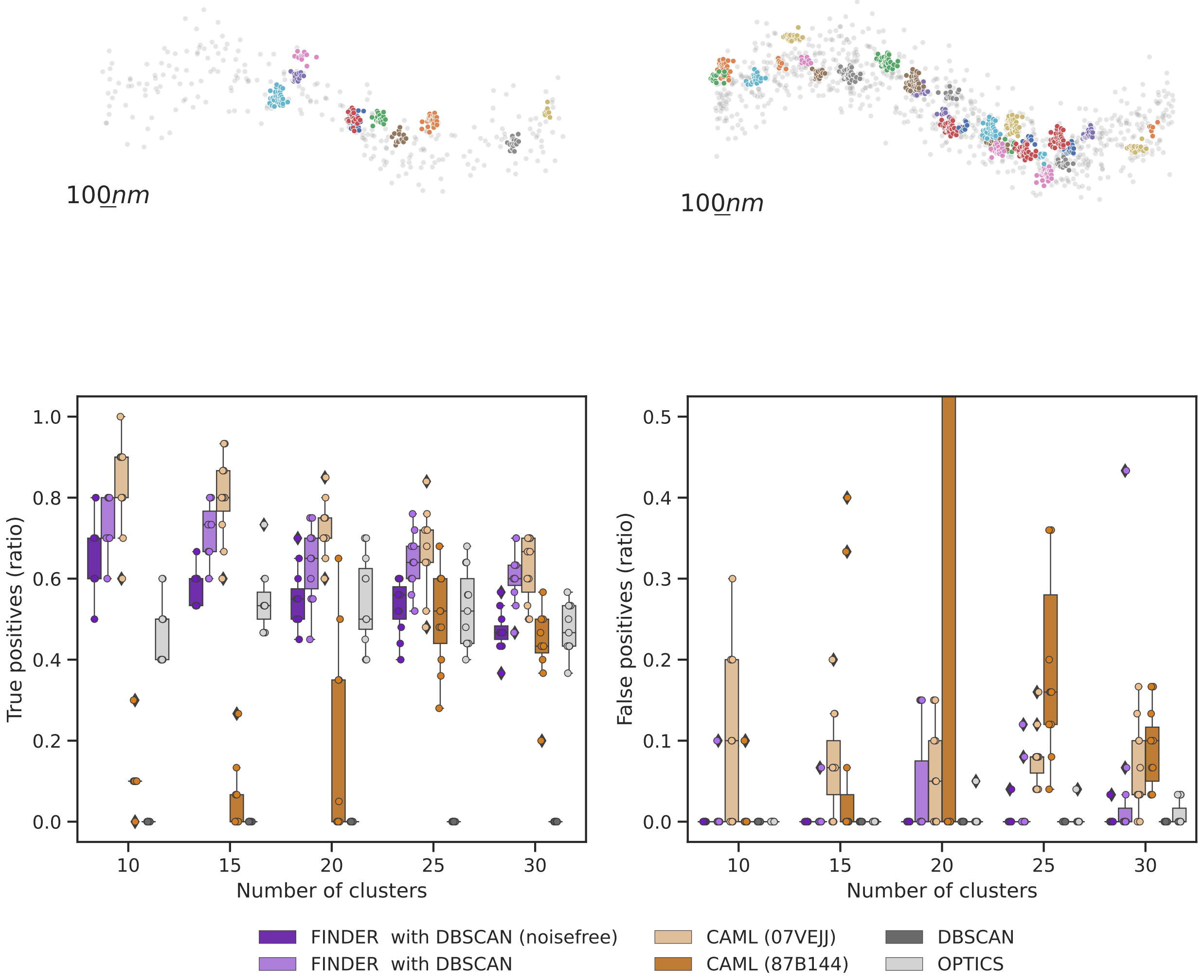


Figure S8: Relates to Fig. 4 c of the main manuscript. Clusters from a SMLM dataset of a synapse [9] (see Fig. 1(a)) are randomly assigned to a sinusoidal path. Based on the number of localizations of the added clusters, a ratio of 1.5 of random noise localizations was then added to the domain. The number of clusters added to the domain was then increased from 10 to 30, therefore increasing noise and overlap. See caption of Fig. S6 for details.

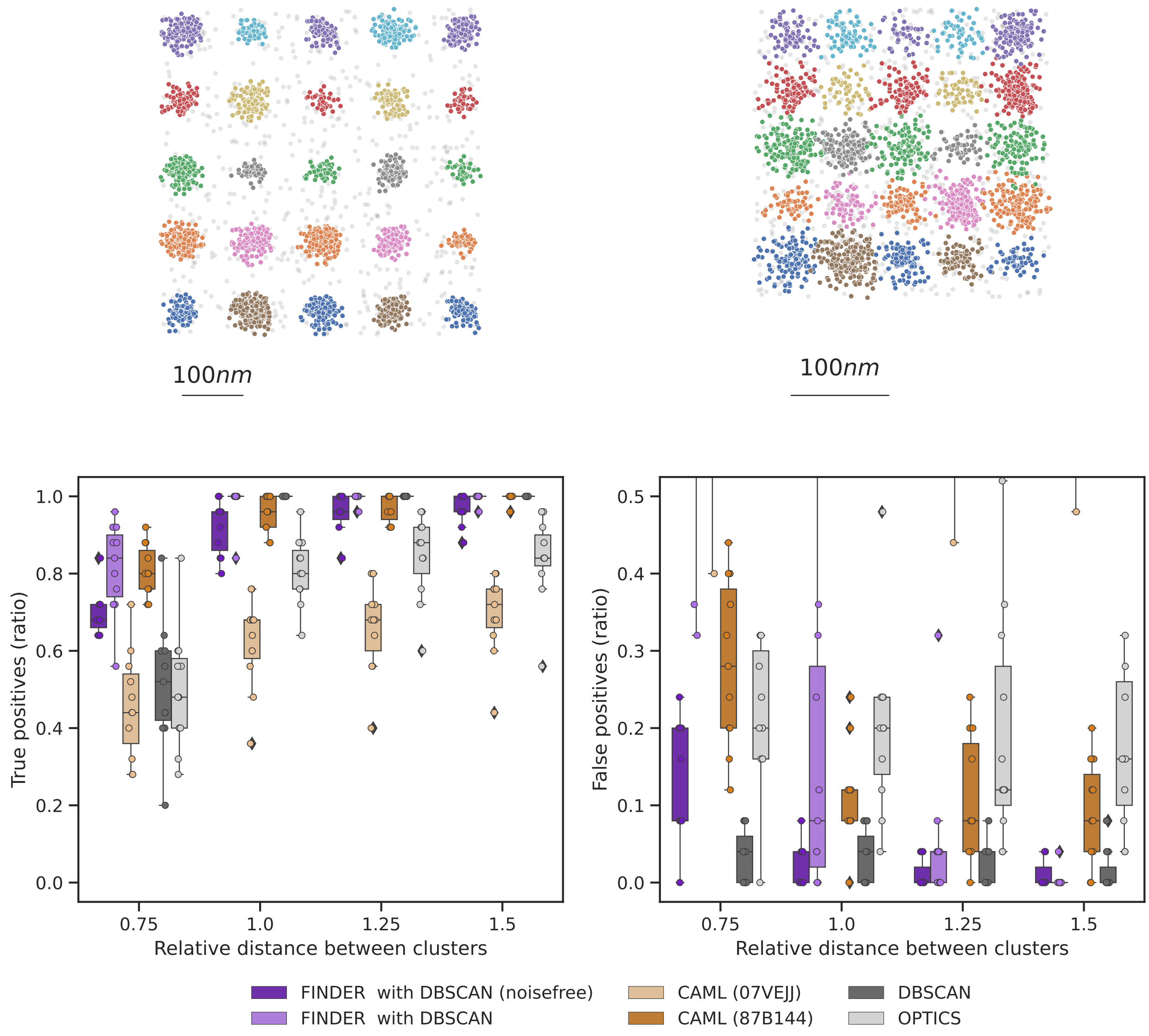


Figure S9: Relates to Fig. 4 d of the main manuscript. Clusters from a DNA origami dataset (see Fig. 1(b)) are randomly assigned to a $5 \times 5$ grid, with spacing $115\mu$m. Based on the number of localizations, an increasing ratio of random noise localizations was then added to the domain. See caption of Fig. S6 for details.

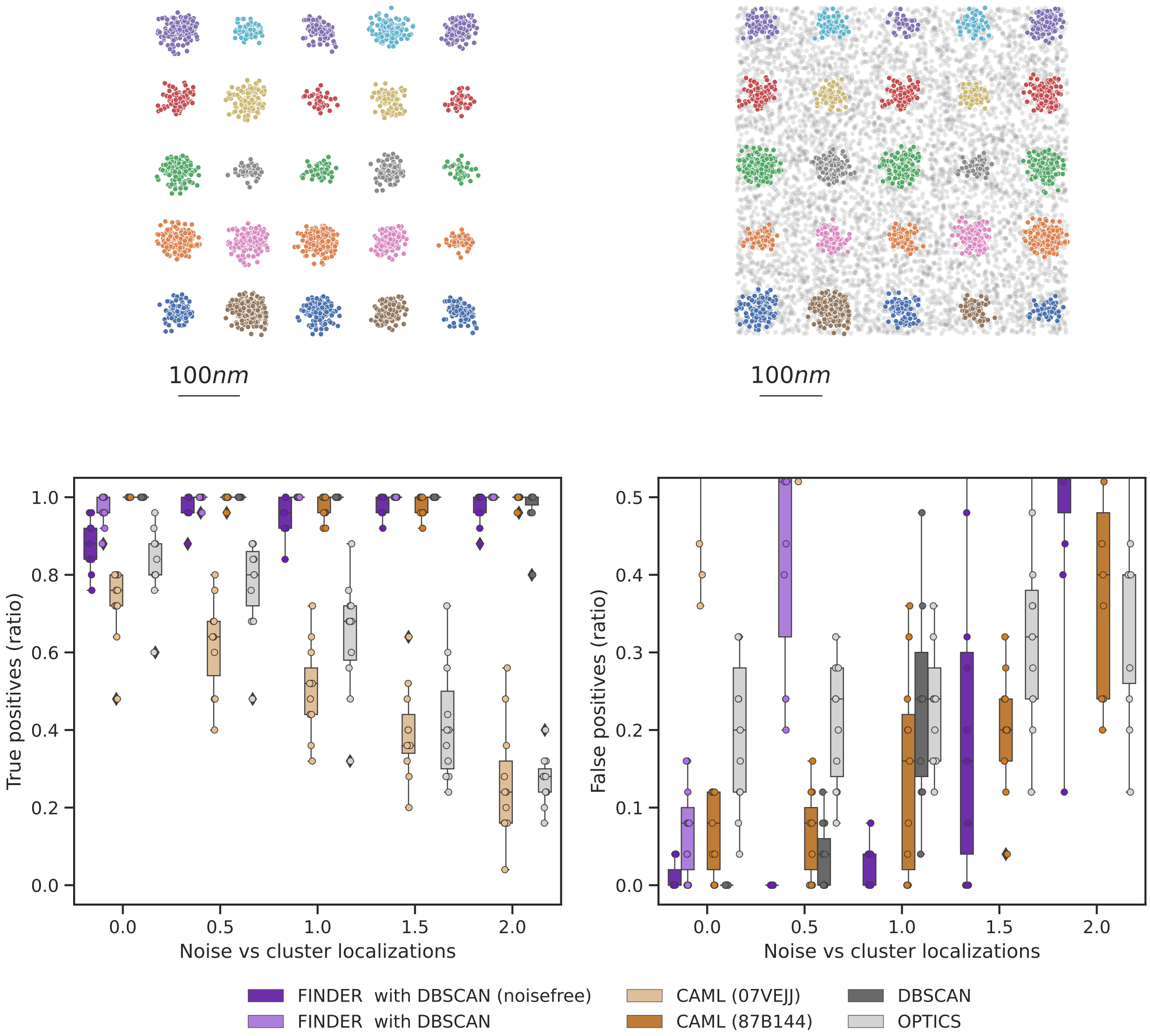


Figure S10: Relates to Fig. 4 e of the main manuscript. Clusters from a DNA origami dataset (see Fig. 1(b)) are randomly assigned to a $5 \times 5$ grid. Based on the number of localizations, a ratio of 0.2 of random noise localizations was then added to the domain. The spacing in between clusters was decreased, here shown as multiples of the maximal cluster diameter of the cluster library, here $77nm$. See caption of Fig. S6 for details.

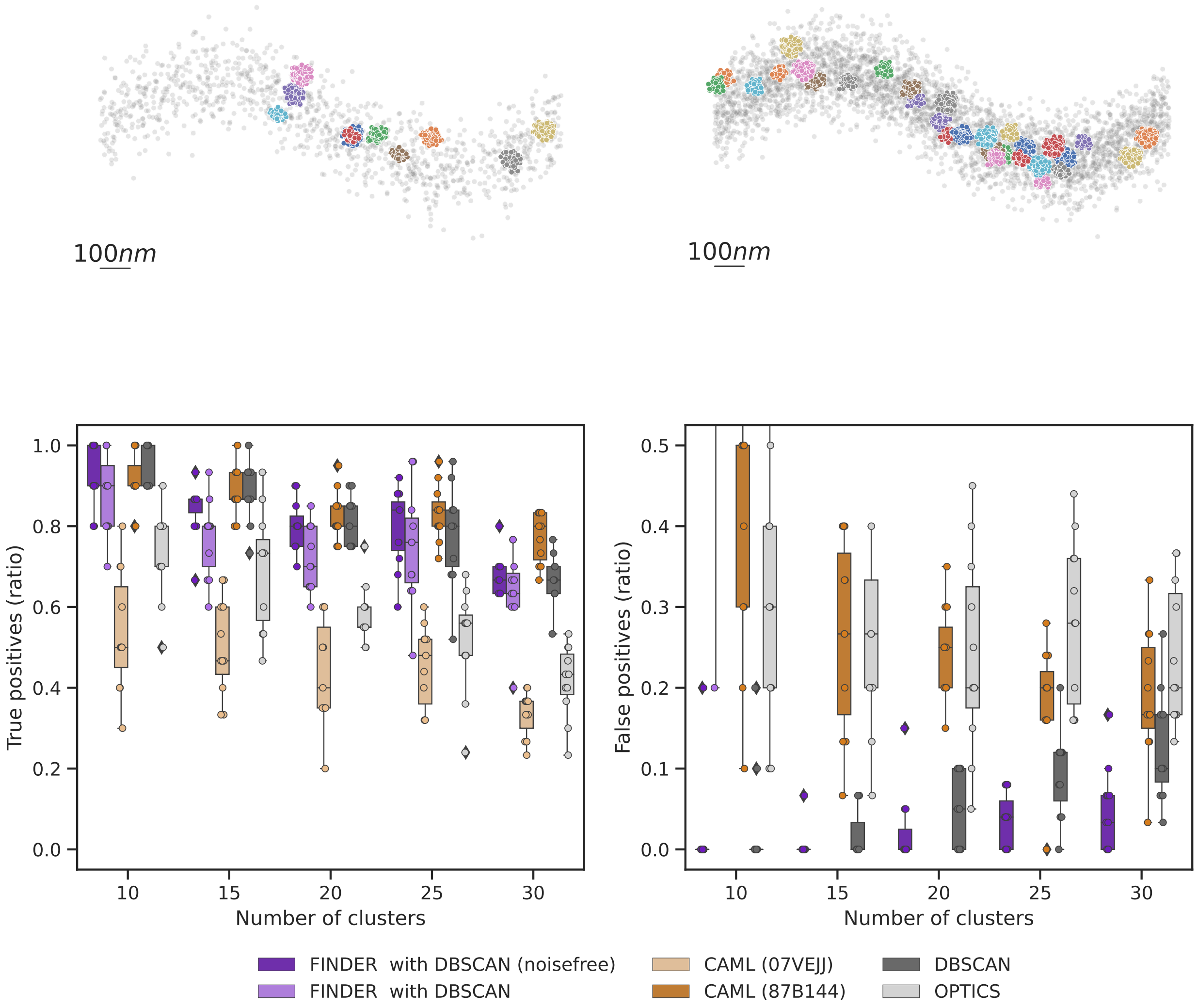


Figure S11: Relates to Fig. 4 f of the main manuscript. Clusters from a DNA origami dataset (see Fig. 1(b)) are randomly assigned to a sinusoidal path. Based on the number of localizations of the added clusters, a ratio of 1.0 of random noise localizations was then added to the domain. The number of clusters added to the domain was then increased from 10 to 30, therefore increasing noise and overlap. See caption of Fig. S6 for details.

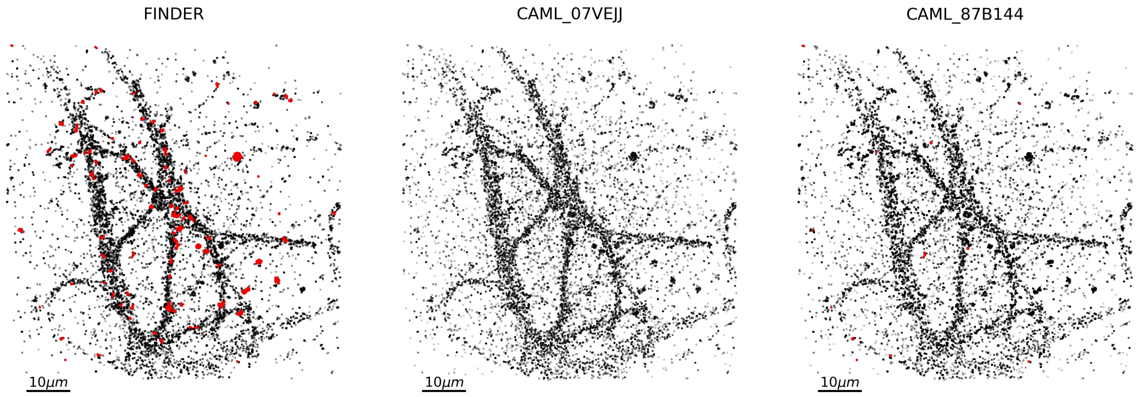


Figure S12: Related to Fig. 5. All localizations not identified as noise. Localizations assigned to clusters of more than 400 are highlighted in red.

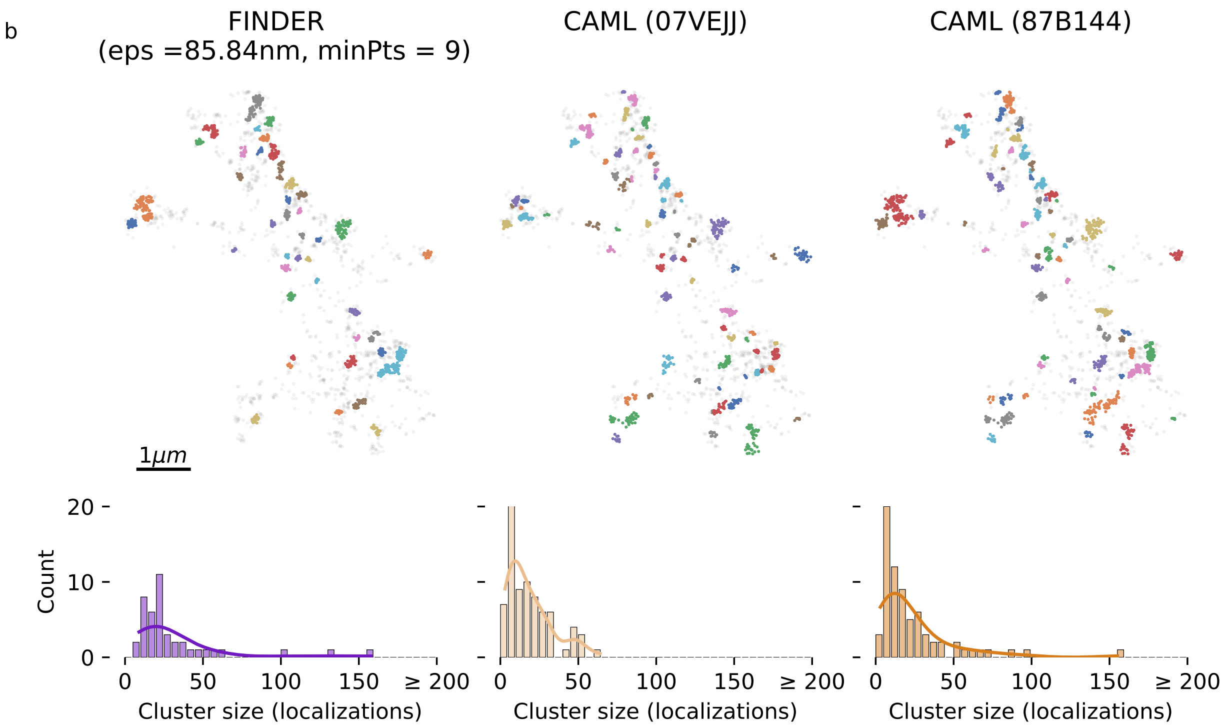


(a) The top row depicts selected clusters (colored points) and localizations that were classified as noise (grey points with transparency). The bottom row shows the distribution of the size of identified clusters.

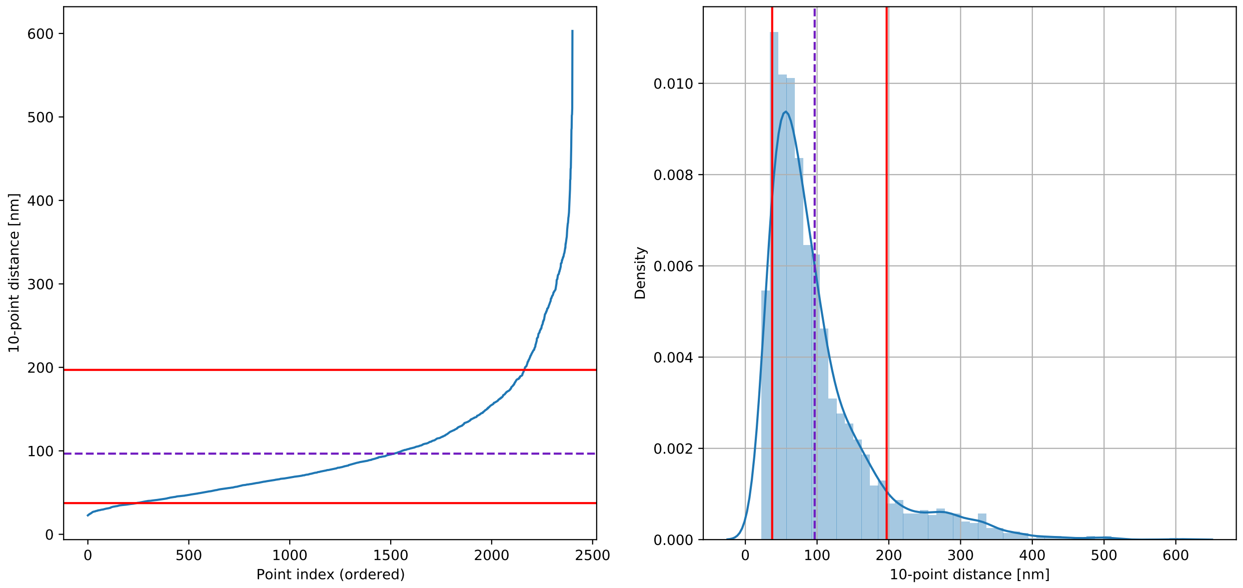


(b) Statistics for the 10th-neighbor distances. Left: Distances for all points, ordered by length. Right: Distribution of distances. The red lines are the 10th and the 90th percentile of the distribution. The violet dashed line denotes the value for $\epsilon$ identified by FINDER.

Figure S13: Analysis analogous to Fig. 5, but for super resolved neuronal AMPA receptor localizations [9]. As in Fig. 5, FINDER identifies fewer clusters with few localizations (cluster size $< 25$). See Fig. S18 for an analysis of the full phasespace.

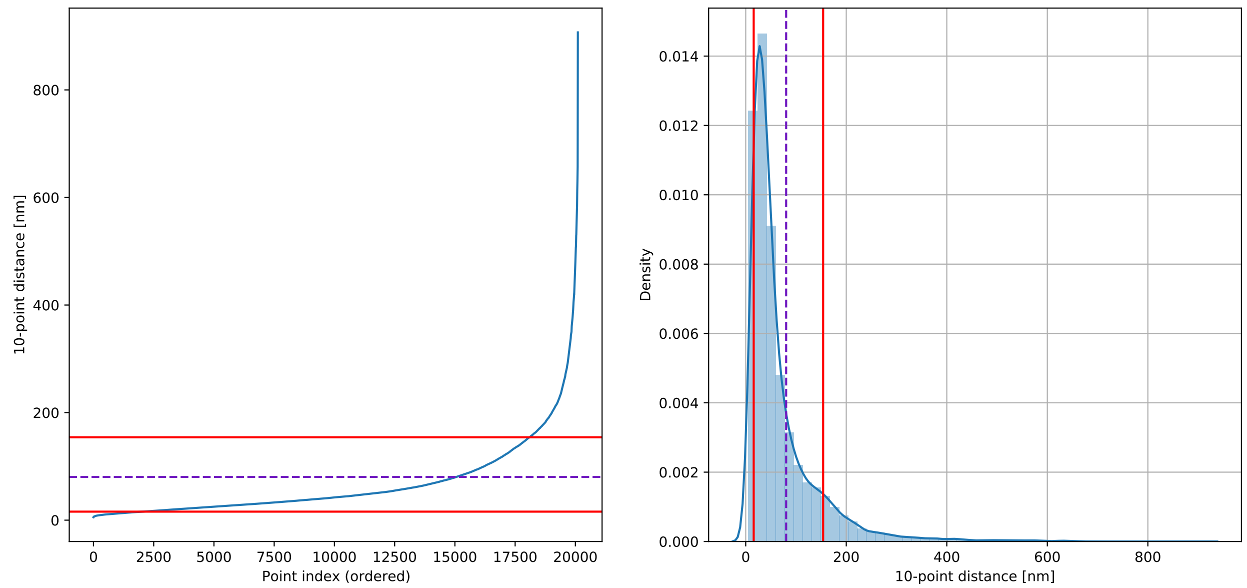


Figure S14: Statistics for the 10th-neighbor distances for the case analyzed in Fig. 5. Left: Distances for all points, ordered by length. Right: Distribution of distances. The red lines are the 10th and the 90th percentile of the distribution. The violet dashed line denotes the optimal value for $\epsilon$ identified by FINDER.

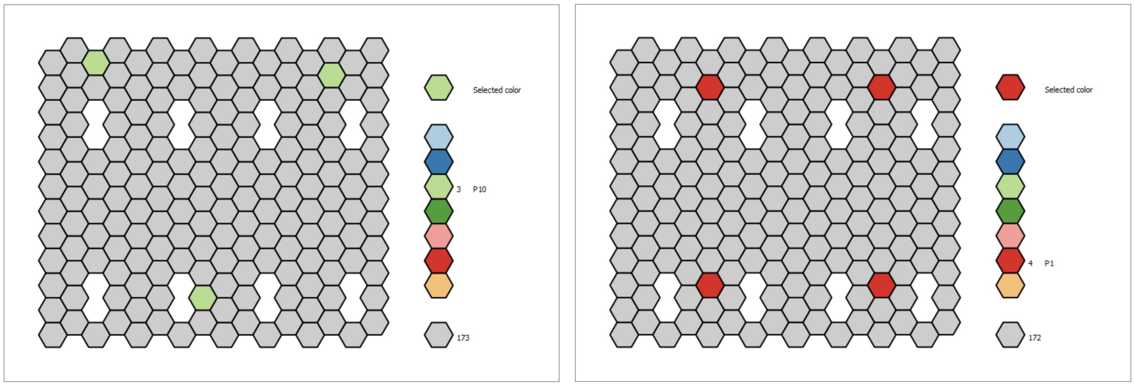


Figure S15: Left: 3-fold symmetric DNA origami containing 3 binding sites. Right: 4-fold symmetric DNA origami containing 4 binding sites.

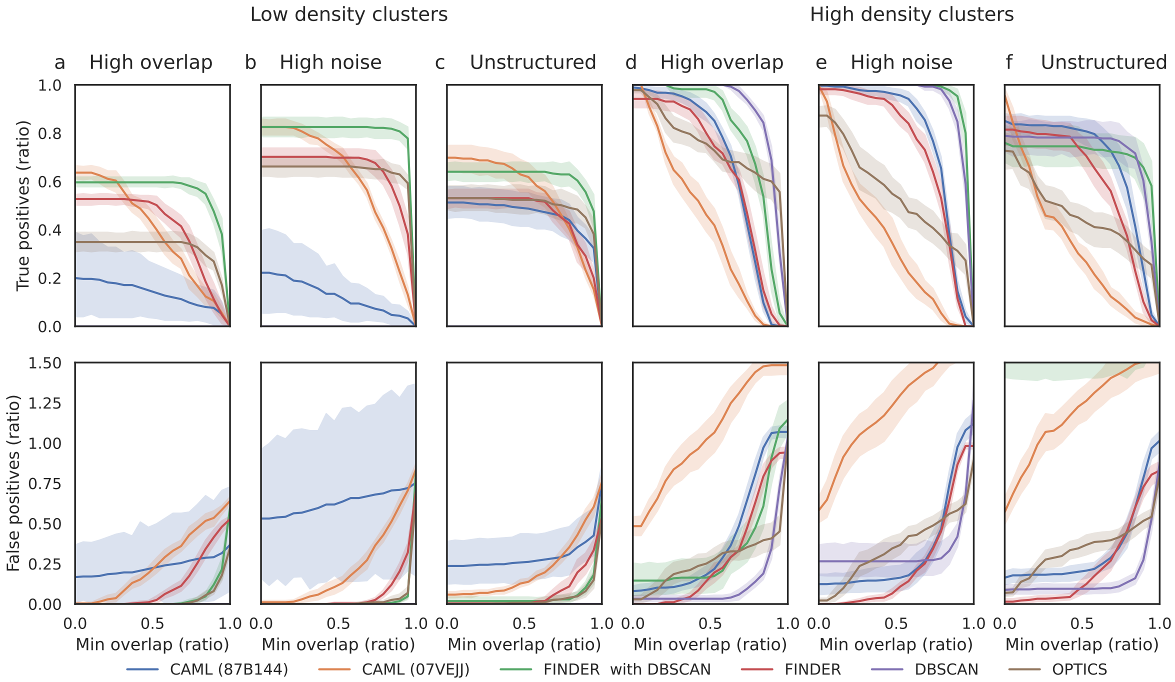


Figure S16: Re-evaluation of the results from Fig. 4. When comparing the clustering outcome to the ground truth, the required overlap between the detected and the ground truth cluster is varied here from 0 to 1 (100%). Note that the clustering outcome does not change with the variation of this threshold – only the ratio of clusters that are attributed to being true or false positives changes. Ratios are given with respect to the number of ground truth clusters.

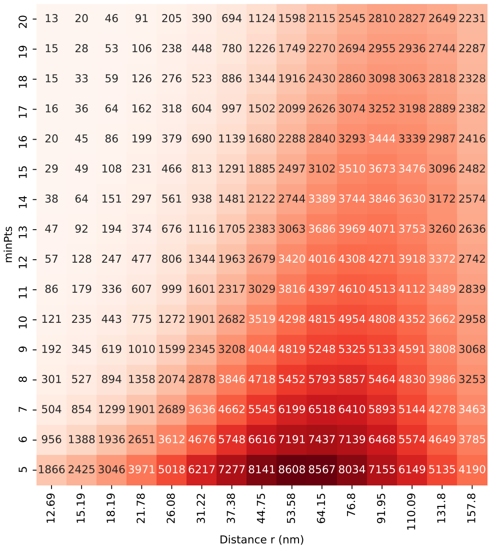


(a) Number of detected clusters, as a function of the two parameters for DBSCAN (noisefree). The distance parameter (x-axis) is logarithmically scaled.

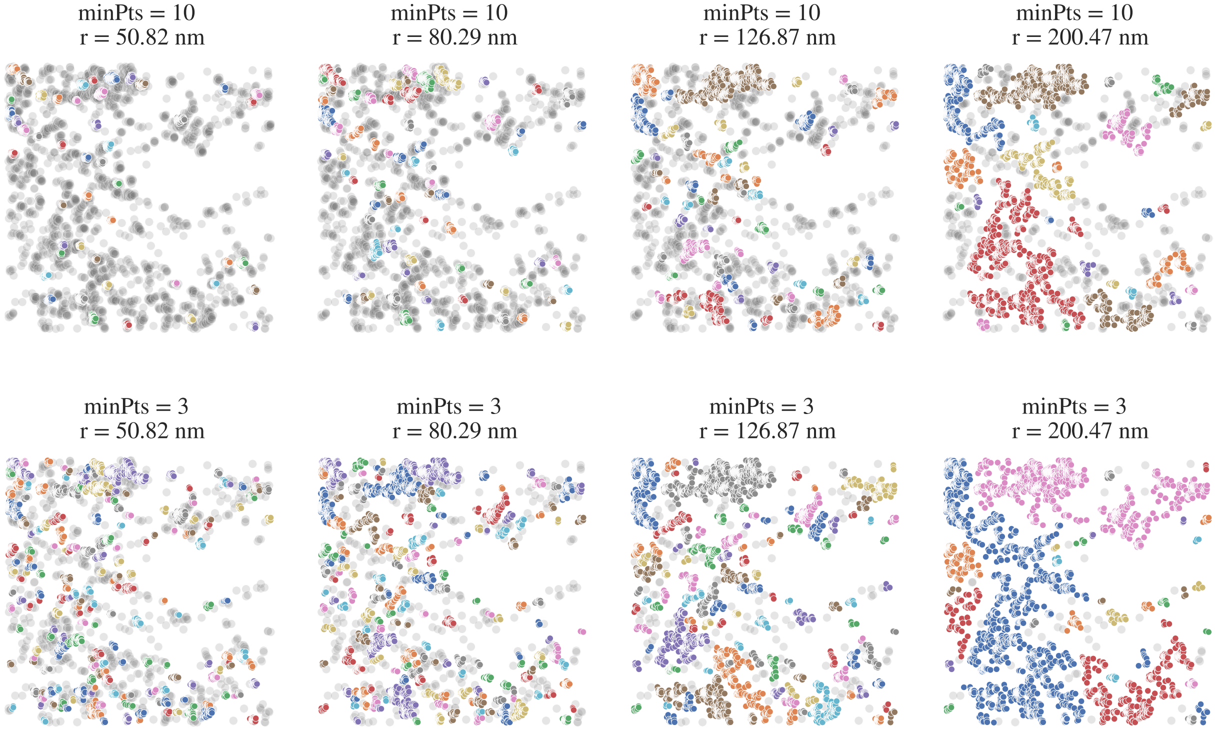


(b) Examples of clustering outcomes for $minPts = 10$ (top row) and $minPts = 3$ (bottom row).

Figure S17: Exploration of clustering outcomes with DBSCAN (noisefree) for the localizations within the red rectangle in Fig. 5. Increasing $minPts$ and decreasing $\varepsilon$ have the effect of excluding less dense – or random – cluster formations and breaking up large clusters. For instance in panel (a) at $\varepsilon = 200nm$, with $minPts = 3$, 34 clusters are detected. Increasing $minPts$ first leads to a decrease of the number of detected clusters (25 clusters for $minPts = 6$), as small, less dense clusters are excluded, and then an increase (30 detected clusters for $minPts = 30$), as larger clusters are broken up. Both effects overlap, and repeat, depending on the statistics of the localizations. In order to exclude the spurious clusters detected at low thresholds, the first steep decrease in cluster densities is excluded by setting $minPts = 10$, see also Fig. S18.

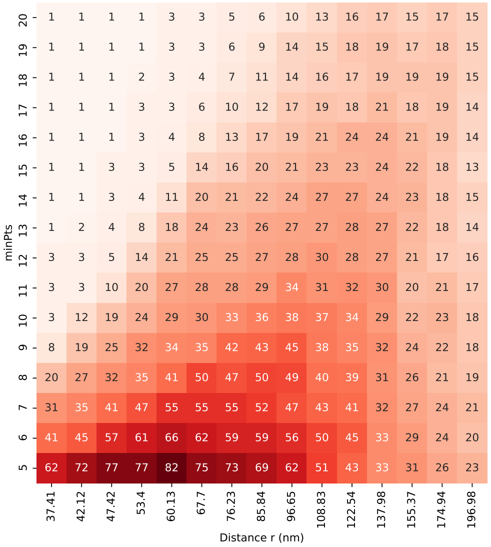


(a) Number of detected clusters, as a function of the two parameters for DBSCAN (noisefree). The distance parameter (x-axis) is logarithmically scaled.

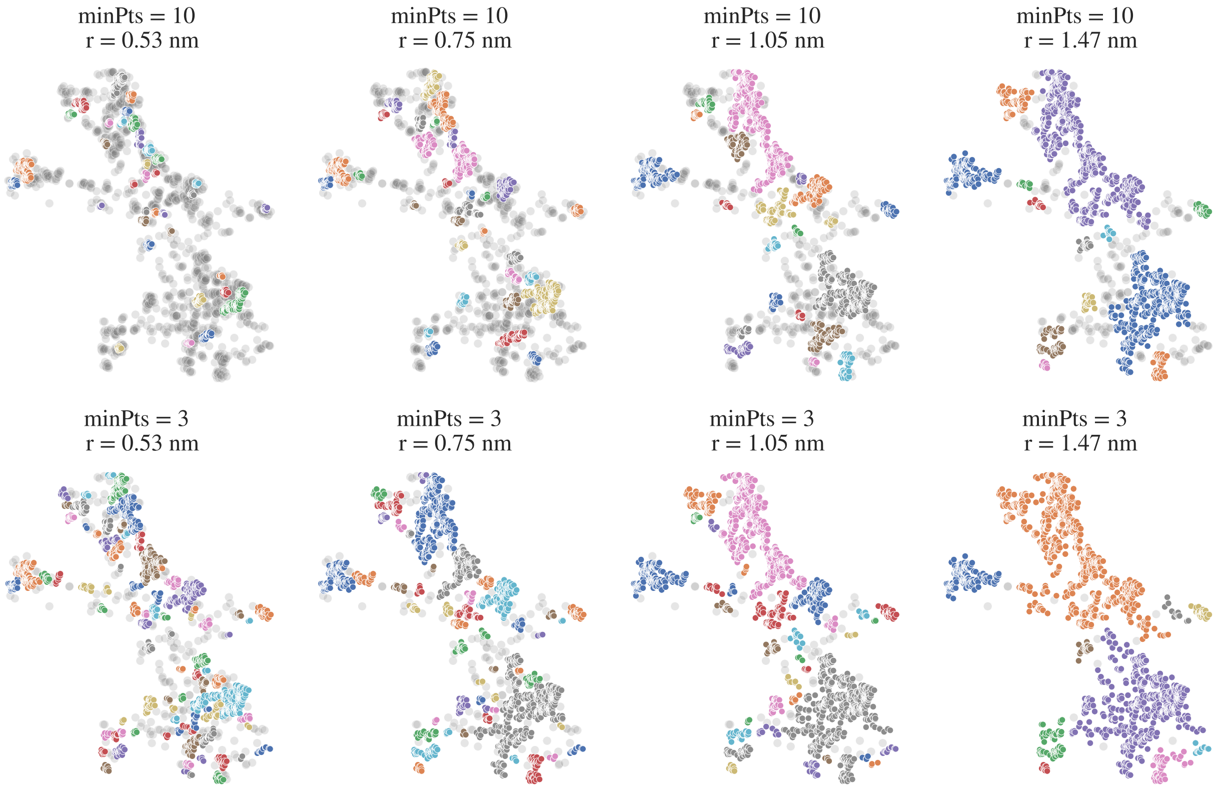


(b) Examples of clustering outcomes for $minPts = 10$ (top row) and $minPts = 3$ (bottom row).

Figure S18: Exploration of clustering outcomes for the localizations in Fig. 5, analysis analogous to Fig. S17.

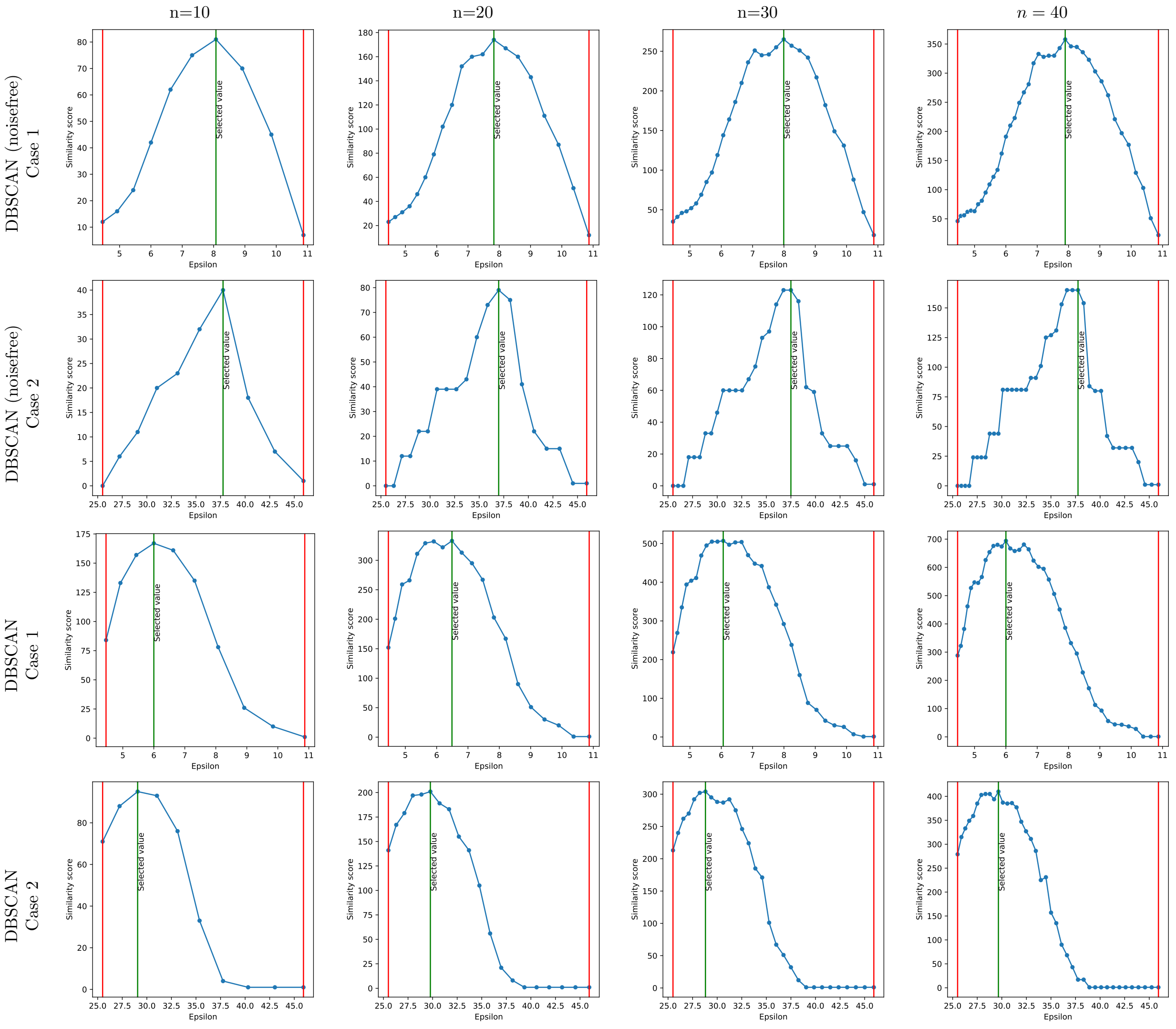


Figure S19: Analysis of examples shown in Figs. S3 and S4, for which the number of $\varepsilon$-values within the domain of interest is varied between 10 and 40. It is seen that the similarity score is robust with respect to s in the number of points.
